# Stag3 regulates microtubule stability to maintain euploidy during mouse oocyte meiotic maturation

**DOI:** 10.18632/oncotarget.13684

**Published:** 2016-11-29

**Authors:** Mianqun Zhang, Xiaoxin Dai, Yalu Sun, Yajuan Lu, Changyin Zhou, Yilong Miao, Ying Wang, Bo Xiong

**Affiliations:** ^1^ College of Animal Science and Technology, Nanjing Agricultural University, Nanjing 210095, China; ^2^ Department of Obstetrics and Gynecology, Peking University Third Hospital, Beijing 100191, China

**Keywords:** Stag3, spindle assembly, chromosome alignment, microtubule stability, aneuploid egg

## Abstract

Stag3, a meiosis-specific subunit of cohesin complex, has been demonstrated to function in both male and female reproductive systems in mammals. However, its roles during oocyte meiotic maturation have not been fully defined. In the present study, we report that Stag3 uniquely accumulates on the spindle apparatus and colocalizes with microtubule fibers during mouse oocyte meiotic maturation. Depletion of Stag3 by gene-targeting morpholino disrupts normal spindle assembly and chromosome alignment in oocytes. We also find that depletion of Stag3 reduces the acetylated level of tubulin and microtubule resistance to microtubule depolymerizing drug, suggesting that Stag3 is required for microtubule stability. Consistent with these observations, kinetochore-microtubule attachment, an important mechanism controlling chromosome alignment, is severely impaired in Stag3-depleted oocytes, resultantly causing the significantly increased incidence of aneuploid eggs. Collectively, our data reveal that Stag3 is a novel regulator of microtubule dynamics to ensure euploidy during moue oocyte meiotic maturation.

## INTRODUCTION

Cells divide and reproduce in two ways: mitosis and meiosis. During mitosis, chromosomes have to replicate and the resulting sister chromatids have to segregate in each cell cycle, which generates two genetically identical daughter cells [1, 2]. On the other hand, male and female germ cells both undergo two rounds of meiotic cell divisions (meiosis I and II) during their development in order to reduce the ploidy, and this process generates the formation of haploid gametes, each possessing half the number of chromosomes of the original cells [3, 4]. Errors in meiosis resulting in aneuploidy contribute to miscarriage, the age-related infertility, and the high incidence of genetic disorders such as Down's syndrome in humans [5, 6, 7, 8].

The major causes of these errors are mainly due to the deficient structure of spindles and segregation of chromosomes [9]. Correct spindle assembly depends on the speedy reorganization of dynamic microtubules which are composed of α- and β-tubulin dimers that make a highly dynamic state between rapid growth and shrinkage. Disruption of this dynamics would cause increased or decreased microtubule stability, which thereby results in defective spindle structures [10]. Faithful chromosome segregation depends on the sister chromatid cohesion mediated by a complex named cohesin, which has the ability to hold two DNA segments together within its ring-shaped structure until the onset of anaphase, when cohesin is cleaved and chromosome segregation is initiated [11–13]. Although numerous studies have been focused on the mechanisms regarding how cohesin and its regulators play essential roles in chromosome segregation, transcriptional regulation and DNA damage repair, the more biological processes involved remain to be explored.

Stag3, Rec8 and Smc1β are three core meiosis-specific versions of cohesin subunits, which are essential for proper pairing and segregation of chromosomes in meiosis [14, 15, 16]. Recent studies have shown that Stag3 is involved in maintaining the stability of all meiosis-specific cohesin complexes, and changing the structures of chromosomes that mediate chromosome pairing and synapsis, DNA repair and progression of meiosis [17]. Both Stag3-deficient male and female mice are infertile due to the early prophase I arrest and apoptosis in both male and female germ cells. Stag3-deficient females show a lack of ovarian follicles indicating a severe ovarian dysgenesis [18, 19]. In addition, the whole-exome sequencing in a large consanguineous family with inherited premature ovarian failure (POF) has identified a homozygous frameshift mutation in the *STAG3* gene leading to a premature stop codon [20]. Despite the very important recent progress, the functions of Stag3 during mouse oocyte meiotic maturation are still not fully defined.

Here, we identify the meiosis-specific subunit of cohesin complex Stag3 as a novel regulator of microtubule dynamics during mouse oocyte meiotic maturation. We show that Stag3 specifically localizes on the spindle apparatus and is required for microtubule stability and spindle assembly. In addition, Stag3 plays an important role in proper kinetochore-microtubule attachments to maintain the euploidy in the mouse eggs, and this role is beyond the sister chromatid cohesion.

## RESULTS

### Stag3 localizes on the microtubule fibers during mouse oocyte meiosis

**Figure 1:**
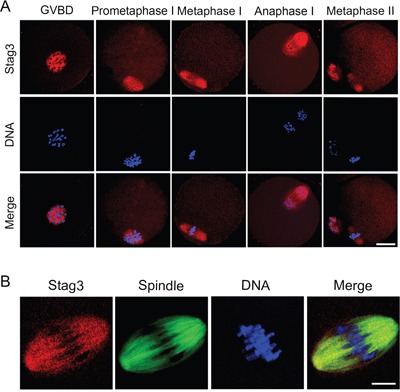
Localization of Stag3 during mouse oocyte meiotic maturation **A.** Mouse oocytes at GVBD, prometaphase I, metaphase I, anaphase I and metaphase II stages were immunolabeled with anti-Stag3 antibody (red) and counterstained with Hoechst (blue). Images were acquired under the confocal microscope. Scale bar, 20 μm. **B.** Metaphase I oocytes were double-stained with anti-Stag3 antibody (red) and anti-α-tubulin-FITC antibody (green) and then counterstained with Hoechst (blue). Scale bar, 10 μm.

The subcellular localization of Stag3 in various developmental stages of mouse oocytes was examined by the immunofluorescent staining. As shown in Figure 1A, in oocytes that just underwent GVBD (Germinal Vesicle Breakdown), with the formation of microtubules, Stag3 started to distribute around the chromosomes (Figure 1A). In metaphase I and metaphase II oocytes, Stag3 exhibited a spindle-like localization pattern (Figure 1A). To confirm the possible relationship between Stag3 and spindle apparatus, we double stained Stag3 with microtubule subunit α-tubulin. As expected, the result showed that fluorescent signals of Stag3 and α-tubulin were indeed overlapped in the metaphase I oocytes (Figure 1B), indicating that Stag3 is colocalized with microtubule fibers during oocyte meiosis.

### Depletion of Stag3 disrupts meiotic spindle assembly and chromosome alignment during mouse oocyte meiosis

**Figure 2:**
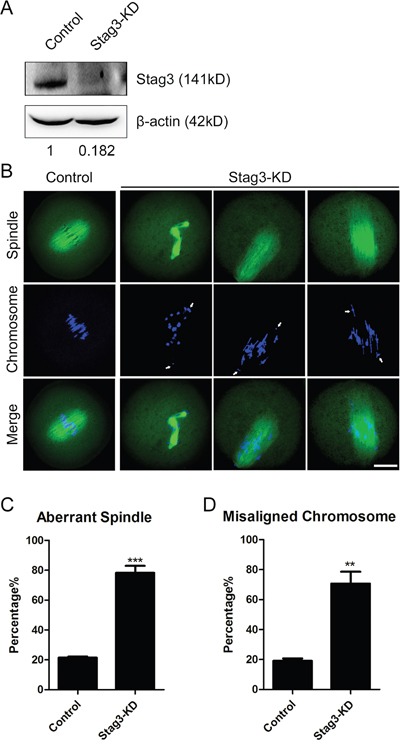
Effects of Stag3 depletion on the spindle formation and chromosome alignment in mouse oocytes **A.** Protein levels of Stag3 in control and Stag3-KD (MO injected) oocytes. The blots were probed with anti-Stag3 antibody and anti-β-actin antibody, respectively. **B.** Representative images of spindle morphologies and chromosome alignment in control and Stag3-KD oocytes. Oocytes were immunostained with anti-α-tubulin-FITC antibody to visualize spindles and counterstained with Hoechst to visualize chromosomes. Scale bar, 20μm. **C.** The rate of aberrant spindles was recorded in control and Stag3-KD oocytes. Data were presented as mean percentage (mean ± SEM) of at least three independent experiments. Asterisk denotes statistical difference at a *p < 0.05* level of significance. **D.** The rate of misaligned chromosomes was recorded in control and Stag3-KD oocytes. Data were presented as mean percentage (mean ± SEM) of at least three independent experiments. Asterisk denotes statistical difference at a *p < 0.05* level of significance.

The spindle localization of Stag3 prompted us to examine its possible function in spindle organization. We applied a morpholino-based gene-silencing approach to deplete Stag3. Fully-grown GV oocytes were microinjected with control and *Stag3*-specific morpholinos and arrested in medium supplemented with milrinone for 20 h, allowing enough time to inhibit mRNA translation. As shown in Figure 2A, the protein level of Stag3 in Stag3-depleted oocytes was remarkably reduced compared to controls, verifying the effect of knockdown by morpholino injection. Then MO-injected oocytes were immunostained with anti-α-tubulin-FITC antibody to observe the spindle morphologies and counterstained with Hoechst 33342 to analyze the chromosome alignment. The results showed that most of control oocytes exhibited a typical barrel-shape spindle apparatus with a well-aligned chromosome on the equatorial plate (Figure 2B). By contrast, a variety of disrupted spindle morphologies and misaligned chromosomes was observed in Stag3 MO-injected oocytes (Figure 2B). Statistically, about 80% of oocytes displayed the disorganized spindles (78.3% ± 4.7%, n=142) and more than 70% of oocytes exhibited misaligned chromosomes (70.7% ± 7.9%, n=142) compared to approximately 20% of defects in controls (21.5% ± 0.7%, n=121; 19.2% ± 1.5%, n=121; *p < 0.05*; Figure 2C, 2D). Therefore, the results suggest that Stag3 is required for spindle assembly and chromosome alignment during mouse oocyte meiotic maturation.

### Depletion of Stag3 compromises the microtubule stability during mouse oocyte meiosis

**Figure 3:**
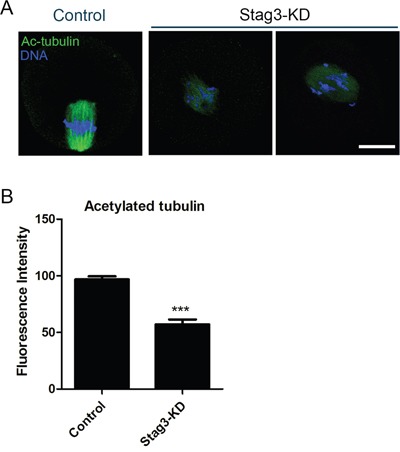
Effects of Stag3 depletion on the acetylated level of α-tubulin in mouse oocytes **A.** Representative images of acetylated α-tubulin in control and Stag3-KD oocytes. Oocytes were immunostained with anti-acetylated α-tubulin antibody and counterstained with Hoechst to visualize chromosomes. Scale bar, 20μm. **B.** The immunofluorescence intensity was recorded in control and Stag3-KD oocytes. Data were presented as mean percentage (mean ± SEM) of at least three independent experiments. Asterisk denotes statistical difference at a *p < 0.05* level of significance.

Impaired spindle assembly predicts that microtubule stability and dynamics might be compromised in the absence of Stag3. To test this, we examined the acetylated level of α-tubulin, a marker of stabilized microtubules, to assess the microtubule stability in oocytes. As shown in Figure 3A, the Stag3-depleted oocytes exhibited a prominently reduced fluorescence intensity of acetylated α-tubulin compared to control oocytes (96.9 ± 2.8, n=45 VS 57.1 ± 4.4, n=39, *p < 0.05*; Figure 3B). The hypoacetylation of α-tubulin suggests that microtubules are less stable with the loss of stag3, which hence impairs microtubule dynamics and organization.

**Figure 4:**
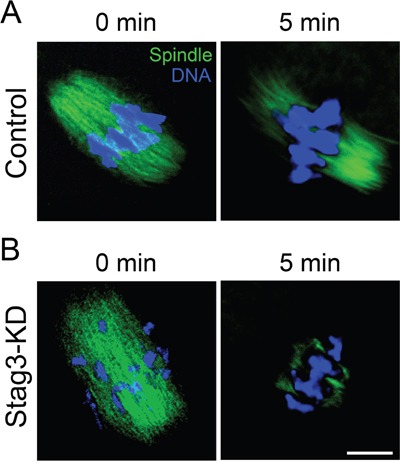
Effects of Stag3 depletion on the microtubule resistance to nocodazole **A.** Representative images of microtubules before and after 5 min of treatment with nocodazole in control oocytes. Oocytes were immunostained with anti-α-tubulin-FITC antibody to visualize microtubules and counterstained with Hoechst to visualize chromosomes. Scale bar, 10 μm. **B.** Representative images of microtubules before and after 5 min of treatment with nocodazole in Stag3-KD oocytes. Oocytes were immunostained with anti-α-tubulin-FITC antibody to visualize microtubules and counterstained with Hoechst to visualize chromosomes. Scale bar, 10 μm.

To further define the role of Stag3 in regulation of microtubule stability, the microtubule resistance to the microtubule depolymerizing drug was tested in the presence of nocodazole. In control oocytes, five minutes after nocodazole treatment, although spindle apparatus was collapsed, microtubules still persisted (Figure 4A). In striking contrast, following the same treatment, microtubules were completely depolymerized in Stag3-depleted oocytes, showing the reduced microtubule stability in these oocytes (Figure 4B). Collectively, these results indicate that Stag3 plays a crucial role in spindle assembly by regulating microtubule stability.

### Depletion of Stag3 impairs kinetochore-microtubule attachments during mouse oocyte meiosis

**Figure 5:**
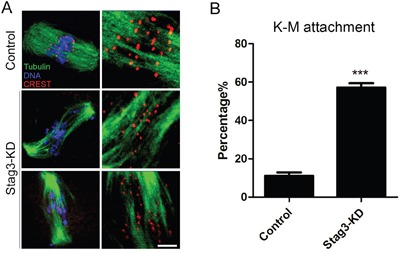
Effects of Stag3 depletion on the kinetochore-microtubule attachment in mouse oocytes **A.** Representative images of kinetochore-microtubule attachments in control and Stag3-KD oocytes. Oocytes were immunostained with anti-α-tubulin-FITC antibody to visualize spindles, with CREST to visualize kinetochores, and counterstained with Hoechst to visualize chromosomes. Scale bar, 10 μm. **B.** The rate of defective kinetochore-microtubule attachments was recorded in control and Stag3-KD oocytes. Data were presented as mean percentage (mean ± SEM) of at least three independent experiments. Asterisk denotes statistical difference at a *p < 0.05* level of significance.

Since aberrant spindle formation and incorrect chromosome alignment is always coupled with the defective interaction between kinetochores and microtubules, we tested the stability of kinetochore-microtubule attachments by employing cold treatment to depolymerize unstable microtubules upon depletion of Stag3. For this purpose, metaphase I oocytes were briefly chilled to induce depolymerization of microtubules that are not attached to kinetochores, and then immunostained with CREST to detect kinetochores, with anti-α-tubulin-FITC antibody to visualize the microtubules and counterstained with Hoechst 33342 to observe chromosomes. It was shown that in a large majority of control oocytes kinetochores were fully attached by microtubules and chromosomes were well-aligned after cold treatment (11.2% ± 1.8%, n=98; Figure 5A, 5B). However, a significantly elevated frequency of kinetochores with very few cold-stable microtubules was observed in Stag3-depleted oocytes (57.2% ± 2.2%, n=100, *p < 0.05*; Figure 5A, 5B). Taken together, this observation suggests that kinetochores and microtubules attach less stably after depletion of Stag3, which might contribute to the failure of chromosome alignment.

### Stag3 is required for maintenance of euploidy in oocytes

**Figure 6:**
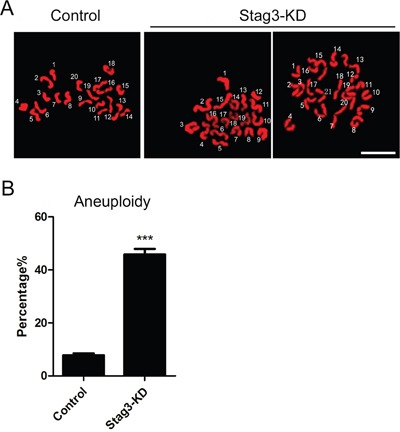
Effects of Stag3 depletion on the generation of aneuploidy in mouse eggs **A.** Representative images of euploid and aneuploid MII eggs. Chromosome spread was performed to count the number of chromosomes. Chromosomes were counterstained with PI. Scale bar, 5μm. **B.** The rate of aneuploid eggs was recorded in control and Stag3-KD oocytes. Data were presented as mean percentage (mean ± SEM) of at least three independent experiments. Asterisk denotes statistical difference at a *p < 0.05* level of significance.

To determine whether chromosome misalignment would result in aneuploidy, an abnormal number of chromosomes in mouse eggs, which might lead to miscarriage, embryonic lethality or genetic disorders, we then analyzed the karyotype of metaphase II oocytes by chromosome spreading. As shown in Figure 6A, the number of single chromosomes (univalents) in the normal eggs was 20, which is expected in the mouse for genomic integrity. Whereas a significantly higher incidence of aneuploid eggs that had more or less 20 univalents was found in Stag3-depleted oocytes compared to controls (7.8% ± 0.7%, n=39 VS 45.8% ± 2.2%, n=31, *p < 0.05*; Figure 6B), suggesting that loss of Stag3 in oocytes are unable to properly assemble the spindles and align the chromosomes and thus prone to produce aneuploid eggs.

## DISCUSSION

*Stag3* encodes a meiosis-specific subunit of the cohesin complex that ensures correct sister chromatid cohesion and enables correct synapsis and segregation of chromosomes during meiosis. Cohesin is the key activity that establishes SCC (Sister Chromatid Cohesion) and then holds sister chromatids until the anaphase. The separation of sister chromatids in mitotic cell division requires the inactivation of SCC function by either proteolytic cleavage or stripping cohesin molecules from chromatin [21–23]. The original (“mitotic” or “somatic”) cohesin complex is postulated to have the shape of a “ring” that is potentially able to physically embrace two chromatids [24].

In mammals there is a meiosis-specific Smc1 subunit (Smc1β), two additional α-kleisins (Rad21L and Rec8) and another stromal antigen protein (Stag3)[25, 26]. All cohesin complexes are composed of these four essential subunits. Only one cohesin component known as Stag3 is represented in all meiosis-specific cohesins. Stag3 is a HEAT repeat protein and it has been reported that Stag3 is involved in segregating sex chromosomes that undergo no recombination [27, 28], which is required for gametogenesis and fertility. Still, we do not know much about the functional roles of Stag3 during oocyte meiotic maturation.

Here, we find that Stag3 accumulates on the spindle apparatus and colocalizes with microtubule fibers in mouse oocytes. This unique localization pattern is different from the typical chromatin distribution of cohesin subunits in mitosis. Also, in meiosis, it has been shown that Stag3 localizes at the interchromatid domain in metaphase I and is lost from chromosome arms during the metaphase-to-anaphase I transition in mouse spermatocytes [29]. Thus, the distinct localization of Stag3 during mouse oocyte meiotic maturation predicts that it might play an important role in microtubule dynamics and spindle organization beyond its function as a subunit of cohesin complex. Consistent with this idea, loss-of-function experiments performed by gene-targeting morpholino micro-injection shows that depletion of Stag3 disrupts the normal spindle formation and correct chromosome alignment with a much higher frequency.

The observation of impaired spindle assembly further prompted us to examine the role of Stag3 in regulation of microtubule dynamics. Tubulin acetylation that occurs on Lys-40 of the α-tubulin subunit has been found in mouse oocytes as an indicator of stabilized microtubules. Acetylated α-tubulin is abundant in stable microtubules but is absent in dynamic subcellular structures [30]. Thus we examined the acetylated level of α-tubulin in Stag3-depleted oocytes. The remarkable reduction in immunofluorescent signals of acetylated α-tubulin indicates that Stag3 is crucial for microtubule stability during mouse oocyte meiosis. Another line of evidence to prove this is that depletion of Stag3 compromises the microtubule resistance to microtubule depolymerizing drug nocodazole. After same time of treatment with nocodazole, microtubule fibers still persist in control oocytes rather than Stag3-depleted oocytes. Taken together, these observations reveal that Stag3 plays an important role in spindle organization through regulating microtubule stability.

The kinetochore contains approximately 100 different proteins that clustered in several different complexes on centromeric DNA, including inner kinetochore proteins, outer kinetochore proteins, as well as regulatory proteins [31, 32, 33]. Through these multiprotein structures, kinetochore attaches chromosomes to spindle microtubules and maintain the attachment to growing or disassembling microtubule to drive chromosomes segregation [34–36]. Therefore, if errors of kinetochore-microtubule attachment could not be corrected until anaphase, they would cause chromosome misalignment and missegregation [37, 38]. We therefore propose that Stag3 participates in maintaining the attachment between kinetochores and microtubules based on the observation of the higher frequency of incorrect chromosome alignment in Stag3-depleted oocytes. As expected, our findings show that a significantly elevated proportion of kinetochores are unattached by microtubules upon cool treatment which is able to depolymerize unattached microtubules. As a result, our data also reveal that loss of Stag3 produces a higher incidence of aneuploid eggs which are highly correlated with miscarriage, birth defects and genetic disorders. The mutations of *TUBB8*, a microtubule related gene, have recently been identified as the genetic cause of human oocyte maturation arrest which occurs frequently in the clinical *in vitro* fertilization, leading to female infertility [39]. However, other undiscovered genes can also result in human oocyte maturation arrest, and our findings suggest that Stag3, as a microtubule regulator, might be one candidate in this pool of genes.

In conclusion, our study presents a body of evidence showing that Stag3 is a master regulator of microtubule dynamics that maintains normal spindle assembly, proper chromosome alignment and correct kinetochore-microtubule attachment to prevent generation of aneuploid eggs, providing a new molecular determinant controlling oocyte development and helping dissect the molecular bases of infertility, miscarriage, early embryonic lethality and birth defects in humans.

## MATERIALS AND METHODS

### Antibodies

Rabbit polyclonal anti-Stag3 antibody were purchased from Cohesion Biosciences (London, UK; Cat#: CPA5158); mouse monoclonal anti-α-tubulin-fluorescein isothiocyanate (FITC) antibody and mouse monoclonal anti-acetyl-tubulin (Lys-40) antibody were purchased from Sigma (St. Louis, MO, USA; Cat#: F2168 and T7451); human anti-centromere antibody was purchased from Antibodies Incorporated (Davis, CA, USA; Cat#: 15-234); FITC-conjugated goat anti-rabbit IgG (H + L), TRITC-conjugated goat anti-rabbit IgG (H + L) and TRITC-conjugated goat anti-mouse IgG (H + L) were purchased from Zhongshan Golden Bridge Biotechnology Co., LTD (Beijing, China).

### Oocyte collection and culture

All experiments were approved by the Animal Care and Use Committee of Nanjing Agricultural University, China and were performed in accordance with institutional guidelines. Female ICR mice (4–6 weeks) were sacrificed by cervical dislocation after intraperitoneal injections of 5 IU pregnant mare serum gonadotropin (PMSG) for 46 h. Fully-grown oocytes arrested at prophase of meiosis I were collected from ovaries in M2 medium (Sigma, St. Louis, MO, USA). Only those immature oocytes displaying a germinal vesicle (GV) were cultured further in M16 medium (Sigma, St. Louis, MO, USA) under liquid paraffin oil at 37°C in an atmosphere of 5% CO2 incubator for *in vitro* maturation. At different time points after culture, oocytes were collected for subsequent analysis.

### Morpholino knockdown

Fully grown GV-intact oocytes were microinjected with 5–10 pl of non-targeting or Stag3-targeting morpholinos (Gene tools, Philomath, OR, USA) in M2 medium containing 2.5 μM milrinone. The working concentration of morpholinos was 1 mM. To facilitate the inhibition of mRNA translation by morpholinos, microinjected oocytes were arrested at GV stage in M16 medium containing 2.5 μM milrinone for 20 h, and then transferred to milrinone-free M16 medium to resume the meiosis for further experiments. Stag3 morpholino sequences: 5′-AATCCAGCTTTAGATGAACACGGCT-3′.

### Immunofluorescence and confocal microscopy

Oocytes were fixed in 4% paraformaldehyde in PBS (pH 7.4) for 30 minutes and permeabilized in 0.5% Triton-X-100 for 20 min at room temperature. Then, oocytes were blocked with 1% BSA-supplemented PBS for 1 h and incubated with anti-Stag3 (1:50), anti-acetyl-tubulin (Lys-40) (1:100), anti-α-tubulin-FITC (1:300), or anti-centromere (1:200) antibodies at 4°C overnight. After washing four times (5 min each) in PBS containing 1% Tween 20 and 0.01% Triton-X 100, oocytes were incubated with an appropriate secondary antibody for 1 h at room temperature. After washing three times, oocytes were counterstained with PI (Propidium Iodide) or Hoechst 33342 (10 μg/ml) for 10 min. Finally, oocytes were mounted on glass slides and observed under a confocal laser scanning microscope (Carl Zeiss 700).

For measurement of immunofluorescent intensity, the signals from both control and experimental oocytes were acquired by performing the same immunostaining procedure and setting up the same parameters of confocal microscope. Data were analyzed by Image J software.

### Western blotting

A pool of 300 oocytes was lysed in 4 × LDS sample buffer (ThermoFisher, Waltham, MA, USA) containing protease inhibitor, and then separated on 10% Bis-Tris precast gels and transferred onto PVDF membranes. The blots were blocked in TBST (Tris-buffred saline containing 0.1% Tween 20) containing 5% low fat dry milk for 1 h at room temperature and then incubated with anti-Stag3 antibody (1:1000) overnight at 4°C. After three times of washes in TBST, the blots were incubated with 1:10,000 dilution of HRP (Horse Radish Peroxidase) conjugated secondary antibodies for 1 h at room temperature. Chemiluminescence was detected with ECL Plus Western Blotting Detection System (GE, Piscataway, NJ, USA) and protein bands were visualized by Tanon-3900. The blots were then stripped and reblotted with anti-β-actin antibody (1:5000) for loading control.

### Chromosome spread

Oocytes were exposed to Tyrode's buffer (pH 2.5) for about 30 s at 37°C to remove zona pellucidae. After recovery in M2 medium for 10 min, oocytes were fixed in a drop of 1% paraformaldehyde with 0.15% Triton X-100 on a glass slide. After air drying, chromosomes were counterstained with PI and examined under a laser scanning confocal microscope.

### Statistical analysis

The data were expressed as mean ± SEM and analyzed by one-way ANOVA, followed by LSD's post hoc test, which was provided by SPSS16.0 statistical software. The level of significance was accepted as *p < 0.05*.
